# Supplementary material for: Blue emission at atomically sharp 1D heterojunctions between graphene and h-BN
Source: Nat Commun. 2020 Oct 23;11:5359. doi: 10.1038/s41467-020-19181-2 (PMC7585426; doi:10.1038/s41467-020-19181-2)
Supplement: Supplementary file 1 — Supplementary Information [file 41467_2020_19181_MOESM1_ESM.pdf]

Blue Emission at Atomically Sharp 1D Heterojunctions between Graphene and h-BN

Gwangwoo Kim[1,11], Kyung Yeol Ma[2,11], Minsu Park[3], Minsu Kim[1], Jonghyuk Jeon[4], Jinouk Song[5], José Eduardo Barrios-Vargas[6], Yuta Sato[7], Yung-Chang Lin[7], Kazu Suenaga[7], Stephan Roche[8,9], Seunghyup Yoo[5], Byeong-Hyeok Sohn[4], Seokwoo Jeon[3] and Hyeon Suk Shin[1,2,10,*]

[1]Department of Chemistry, Ulsan National Institute of Science and Technology (UNIST), Ulsan 44919, Republic of Korea

[2]Department of Energy Engineering, Ulsan National Institute of Science and Technology (UNIST), Ulsan 44919, Republic of Korea

[3]Department of Materials Science and Engineering, Korea Advanced Institute of Science and Technology (KAIST), Daejeon 34141, Republic of Korea

[4]Department of Chemistry, Seoul National University, Seoul 08826, Republic of Korea

[5]School of Electrical Engineering, Korea Advanced Institute of Science and Technology (KAIST), Daejeon 34141, Republic of Korea

[6]Departamento de Física y Química Teórica, Facultad de Química, UNAM, 04510 México City, México

[7]Nanomaterials Research Institute, National Institute of Advanced Industrial Science and Technology (AIST), 1-1-1 Higashi, Tsukuba 305-8565, Japan

[8]Catalan Institute of Nanoscience and Nanotechnology (ICN2), CSIC and The Barcelona Institute of Science and Technology, Campus UAB, Barcelona 08193, Spain

[9]ICREA—Institució Catalana de Recerca i Estudis Avançats, 08010 Barcelona, Spain

[10]Low Dimensional Carbon Material Center, Ulsan National Institute of Science and Technology (UNIST), Ulsan 44919, Republic of Korea

[11]These authors contributed equally: Gwangwoo Kim and Kyung Yeol Ma.

[*]E-mail: shin@unist.ac.kr (H.S.S.)

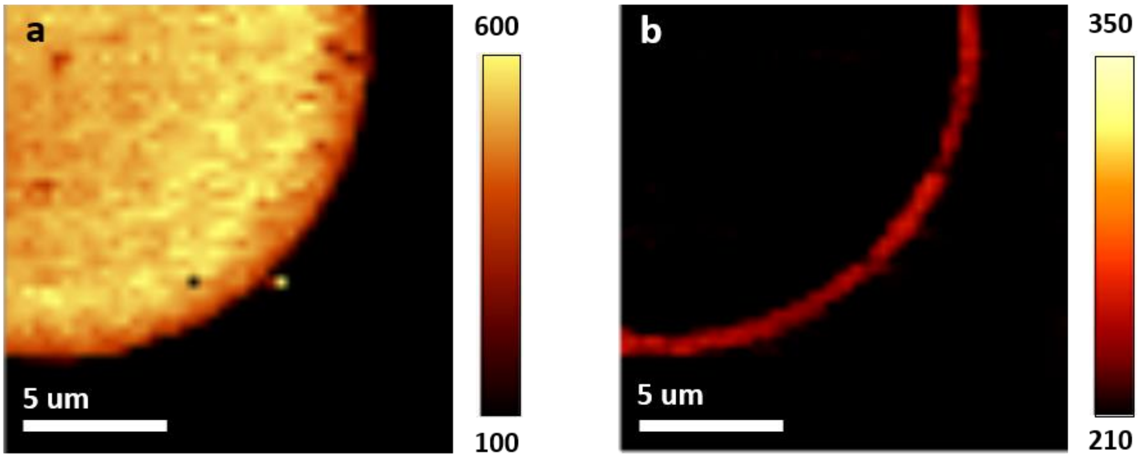

Supplementary Figure 1 | Magnified Raman and PL images of graphene/h-BN in-plane heterostructures. a, **b**, Raman (for 2D band) and PL mapping (at 410 nm) images magnified in Fig. 1e and 1f.

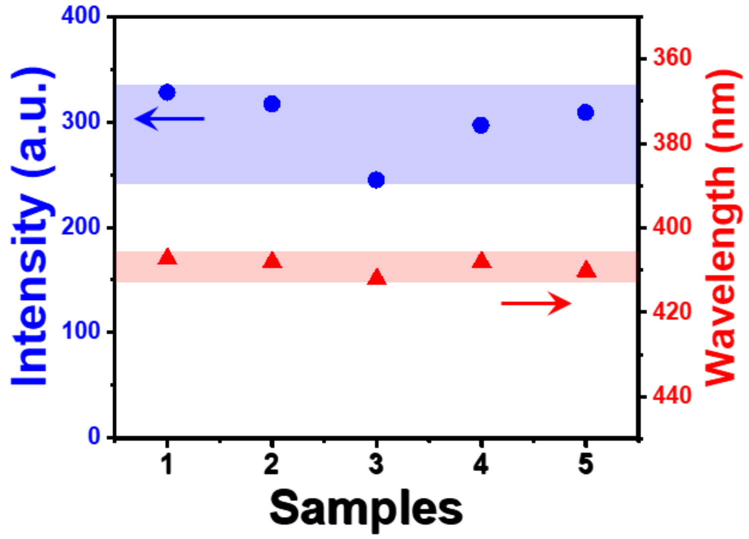

Supplementary Figure 2 | Comparison of PL intensity and wavelength on the different five GQD/h-BN samples. The mean and standard deviation of PL intensity and peak position were 299.2 ± 32.34 and 409.16 ± 1.94 nm, respectively.

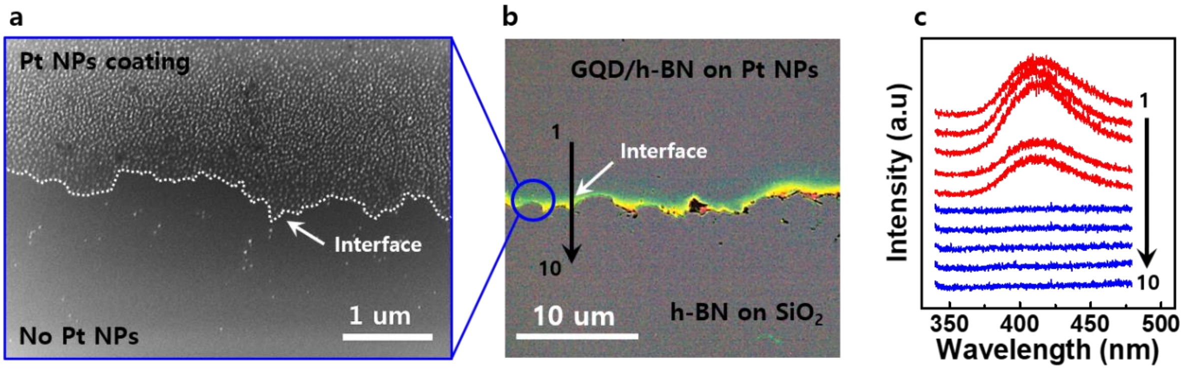

Supplementary Figure 3 | PL analysis after conversion reaction of h-BN on Pt NPs array/SiO_2 and on just SiO_2 substrate. a, SEM image of half-patterned Pt NPs array on the SiO_2/Si substrate. The interface between the regions with and without Pt NPs coating is marked with white line. **b**,**c**, Optical image (b) and PL spectra (c) of GQD/h-BN fabricated on half-patterned Pt NPs/SiO_2 substrate. The positions of spectra (1~10) are marked with black line in (b). The blue spectra in (c) show that the conversion reaction of h-BN on just SiO_2 substrate did not occur.

Fabrication of bare GQDs array without h-BN

In order to check PL emission on bare GQD without h-BN, the sample was prepared using a single-layer graphene grown on Pt foil and O_2 plasma etching process, shown in Figure S3a. First, the graphene monolayer grown on the Pt foil is transferred onto a SiO_2/Si substrate by electrochemical delamination method. After aligning the Pt NPs array on the top of graphene by self-assembly process[1], O_2 plasma etching is performed on the sample at 50 W for 1 min, and in the graphene etching process, Pt NPs array is used as a pattern mask. After the reaction, Pt NPs are removed with aqua regia solution.

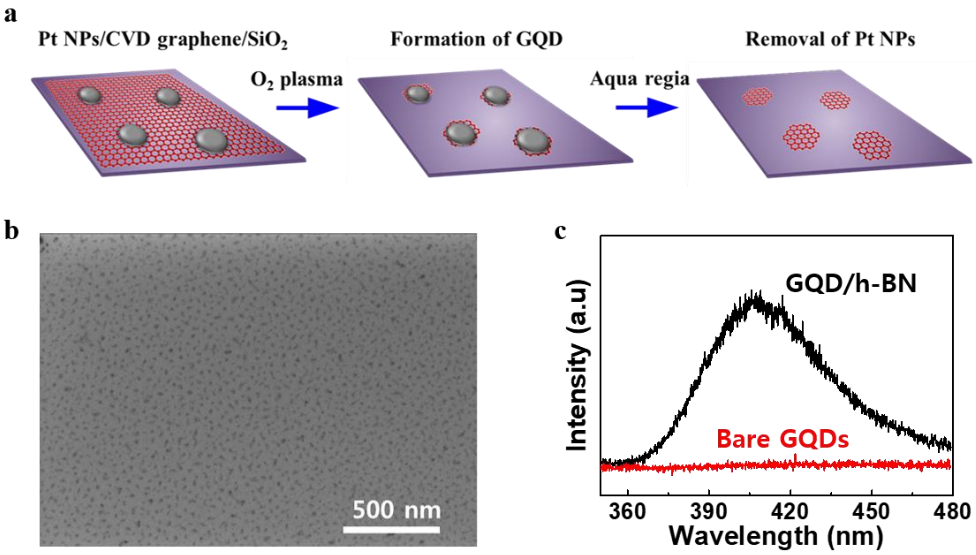

Supplementary Figure 4 | Characterizations of GQDs array without h-BN matrix. a, Scheme of bare GQDs prepared by the O_2 plasma treatment of CVD grown graphene. **b**, SEM image of a bare GQDs (~7 nm) array prepared in (a). **c**, PL spectra of bare GQD (red spectrum).

Fabrication of h-BN sheet with nano-sized holes

The h-BN sheet with nano-sized holes was prepared by using hydrogen-etching of h-BN[2] though the annealing process on Pt NPs in H_2 atmosphere. First, after aligning the Pt NPs array on the SiO_2/Si substrate by self-assembly process[1], a single-layer h-BN CVD-grown on the Pt foil[3] was transferred onto the Pt NPs/SiO_2 substrate by electrochemical delamination method. And then, we carried out hydrogen-etching of h-BN on Pt NPs by annealing at 700 ^{o}C in H_2 flow (30 sccm) for 10 min.

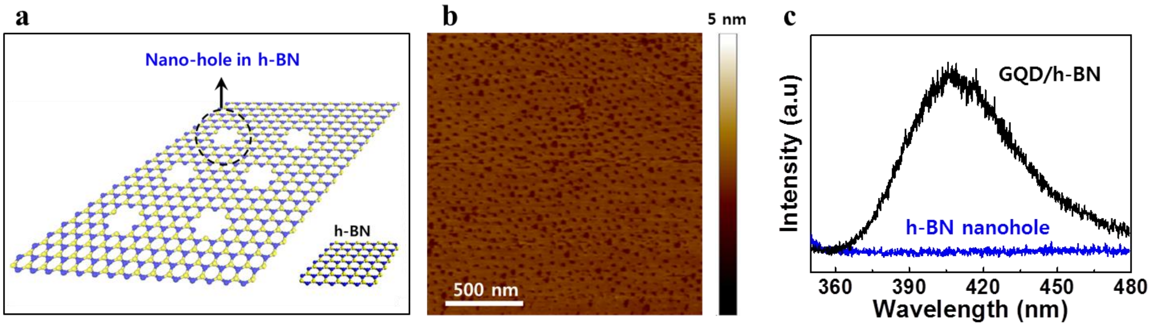

Supplementary Figure 5 | Characterizations of h-BN monolayer with nano-sized holes. a, Scheme of a h-BN sheet with nano-sized holes. **b**, AFM image of h-BN nano-sized holes by annealing process in H_2 atmosphere. **c**, PL spectra of h-BN nano-sized hole (blue spectrum).

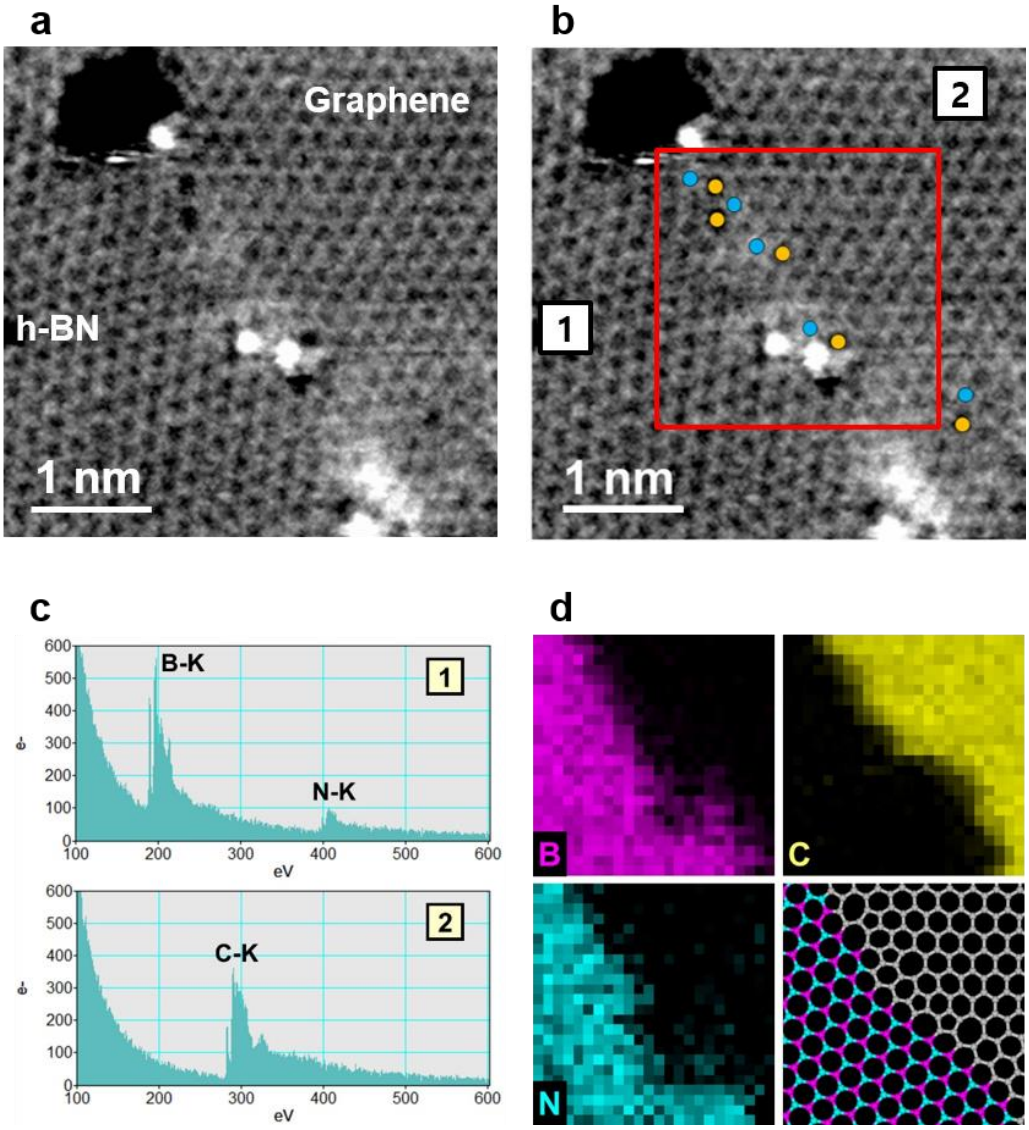

Supplementary Figure 6 | STEM-EELS analysis of h-BN/graphene heterostructure. a, Annular dark-field (ADF) STEM image showing a boundary between h-BN and graphene domains (left and right, respectively). **b,** Magnified ADF-STEM image of the area indicated by a green square in (a). Blue and orange spheres denote 5- and 7-membered rings, respectively. The bright impurity atoms found in the red box of (b) are identified as calcium by EELS, probably coming from the sample transfer process. **c**, EELS data acquired at the positions labeled as #1 and #2 in (a). **d**, STEM-EELS elemental maps of boron, carbon, nitrogen and a possible structure model for the area indicated by a red square in (b).

Theoretical model

In this section, we detail the models of the disordered interfaces between graphene and hBN in-plane heterostructures. Disordered interfaces are found when there is an orientation mismatch between graphene and hBN crystals. Thus, we set-up three different polycrystalline lattices with graphene and hBN crystals of 10 nm, 20 nm and 40 nm average grain size. The polycrystalline samples with average grains size were created using a Voronoi diagram. Each Voronoi cell was filled with randomly oriented honeycomb crystals. In order to achieve a thermodynamically stable structure we annealed the lattice using molecular dynamics as implemented in LAMMPS, with the parameters found in reference.[4]

We calculated the electronic properties using a nearest-neighbor tight-binding Hamiltonian fitted to a Wannierization of DFT calculations.[4] The local density of states projected over all the sites at the graphene-hBN interface (grain boundary sites, GB) was calculated by evaluating the imaginary part of the Green function using the Kernel Polynomial Method with 3000 moments and the Lorentz kernel.[5]

Vertically stacking of GQD/h-BN layers with h-BN intercalation layers

It is known that the stacks of GQDs in the solid state induced photon reabsorption and nonradiative energy transfer which indicates partial PL quenching.[6,7] That is, at the stacked sample, the excited electrons of GQDs may nonradiatively relax to ground states through couplings with neighboring ones (by reabsorption and energy transfer). We solved the issue of nonradiative energy transfer by inserting 3L h-BN as an intercalation layer between GQD/h-BN layers, but the generated photons from GQD/h-BN can be still re-absorbed by adjacent layers. However, note that 3L h-BN as an intercalation layer may not fully prevent the nonradiative energy transfer because charge tunneling through 3L h-BN may occur still. Comparing the PL emission in 1 layer-, 2 layers-, 3 layers-, and 4 layers-stacked GQD/h-BN films with h-BN intercalation layers, the extent of enhanced PL intensity (blue solid line) did not increase by 2, 3, or 4 times (red dotted line) proportionally as shown in Supplementary Fig. S7.

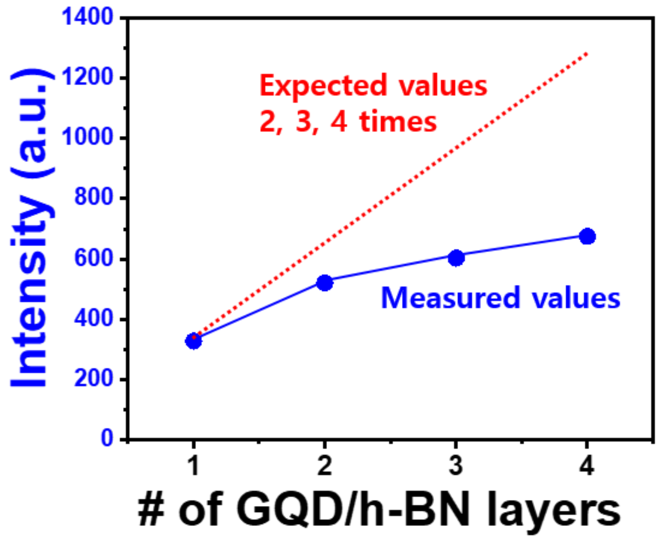

Supplementary Figure 7 | Comparison of PL intensity of the different numbers of GQD/h-BN layers with 3L h-BN as an intercalation layer.

GQD/h-BN layers in an OLED device

The device architecture is shown in Supplementary Fig. S8a, with a simple structure[8,9] consisting of ITO/PEDOT:PSS/PVK/GQD/TPBI/LiF/Al (Supplementary Fig. S9). The PEDOT:PSS, PVK, and TPBI were used as hole injection, hole transport (HTL), and electron transport layers (ETL), respectively. Supplementary Fig. S8b shows the electronic band structure of the device. Note that the reference device is fabricated without GQD/h-BN while having the same structure as above. The current density-voltage curves for two devices of GQD/h-BN and reference (Supplementary Fig. S8c) show that the turn-on voltage decreases from 6 V to 4 V when the GQD/h-BN with G/BN junctions is used as the emitting layer in the device. Moreover, a higher current density at a lower voltage (~5 V) was observed in the device with GQD/h-BN. This indicates that the G/BN junctions in the GQD/h-BN heterostructures may provide additional carrier transport, resulting in enhancement of the overall current density. The normalized EL spectrum (red) of the GQD/h-BN device in Supplementary Fig. S8d shows blue emission with a peak wavelength at 410 nm, which is consistent with the PL spectrum of GQD/h-BN with G/BN junctions (Fig. 2c). However, the reference device without the GQD/h-BN emitting layer showed the broader emission peak at 430 nm (Supplementary Fig. S8d, black spectrum). The external quantum efficiency (EQE) of our best GQD/h-BN device was 0.5% (Supplementary Fig. 10).

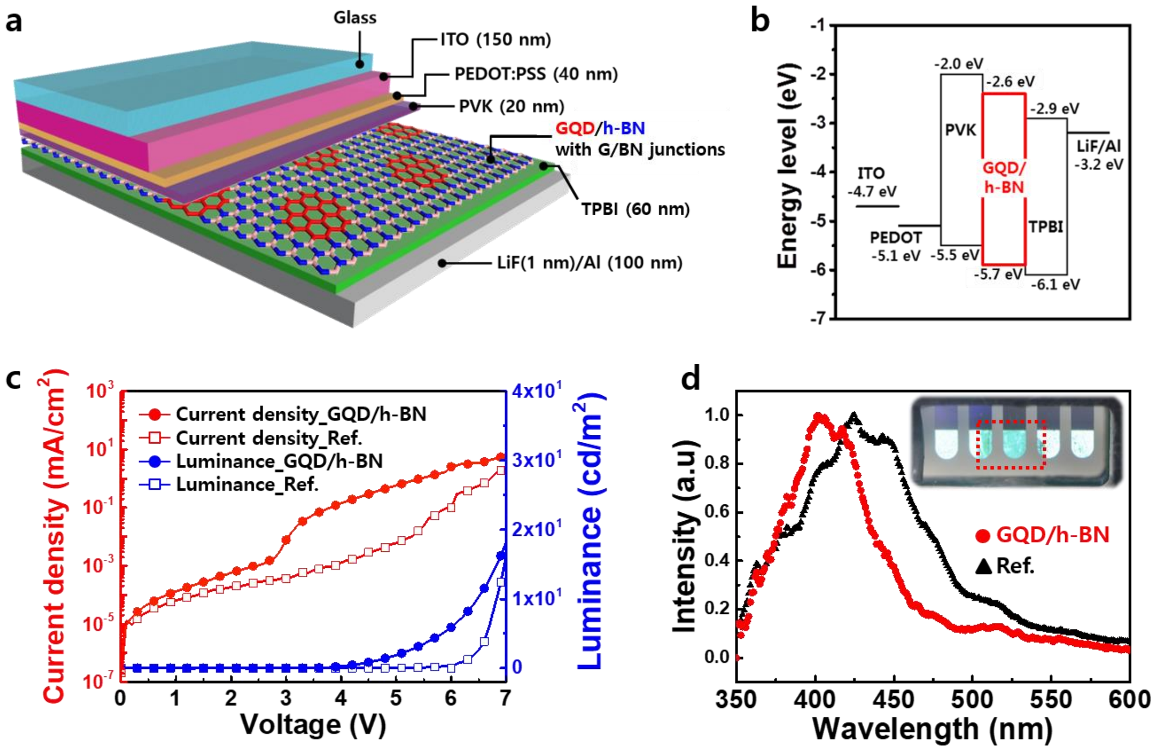

Supplementary Figure 8 | An OLED device using GQD/h-BN layers with G/BN junctions. **a**, Device architecture using GQD/h-BN with G/BN junctions as emitting materials. **b**, Electronic band structure of the device. **c**, Current density-voltage (J-V) and luminance-voltage (L-V) characteristic curves. **d**, EL spectrum for the fabricated GQD/h-BN devices. Inset is the optical image of the LED device with the GQD/h-BN layers. The GQD/h-BN region is marked with a red dotted line.

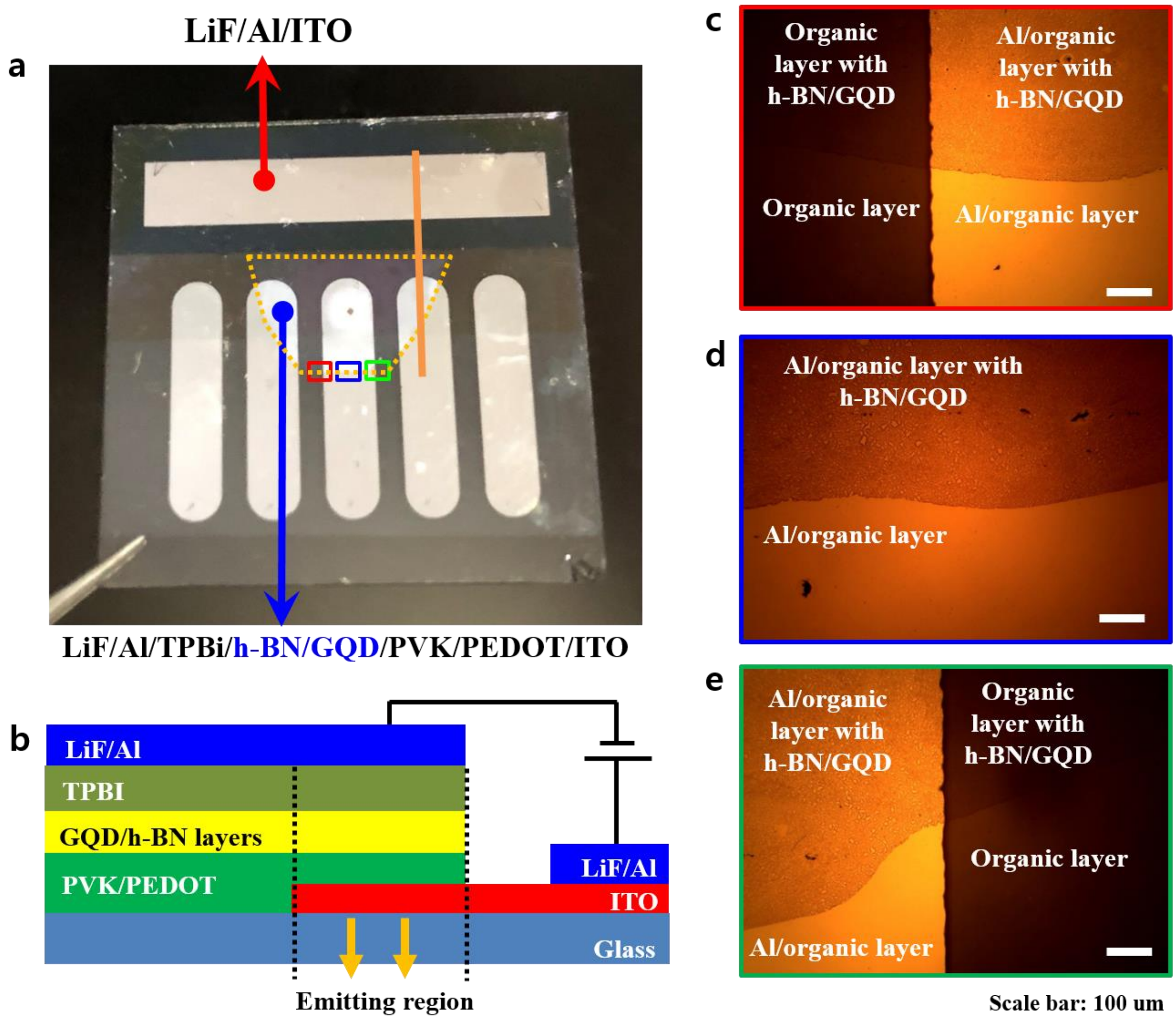

Supplementary Figure 9 | The optical images of the detailed LED device with the GQD/h-BN layers. a, A photograph of organic materials-based LED devices with GQD/h-BN films. The GQD/h-BN region is indicated by a yellow dotted line. **b**, Scheme of cross-sectional device structure marked by orange line in Figure R4a. **c-e**, Enlarged optical microscopic images of each position. (b, red box; c, blue box; d, green box). Scale bars are 100 um.

External Quantum Efficiency (EQE) of the GQD/h-BN devices

The EQE value was calculated by using the following formula.

$$\eta_{EQE} = \frac{Emitted\ photons}{Injected\ Charge} = \frac{Energy\ of\ emitted\ light / Energy\ of\ one\ photon}{Total\ current / Charge\ of\ one\ electron}$$

$$= \frac{\iint_{\lambda,\Omega} \frac{I(\lambda,\Omega)d\lambda(l^2 d\Omega)}{hc/\lambda}}{i_{\text{source}}/e} = \frac{\iint_{\lambda,\theta} \frac{I(\lambda,\theta)d\lambda(2\pi l^2 \sin\theta\, d\theta)}{hc/\lambda}}{i_{\text{source}}/e}$$

$$= \frac{\frac{2\pi l^2}{A_{PD}} \iint_{\lambda,\theta} \frac{\Phi_0(\theta)s(\lambda,\theta)d\lambda \sin\theta\, d\theta}{hc/\lambda}}{i_{source}/e} = \frac{2\pi l^2}{A_{PD}} \frac{e}{i_{source}} \iint_{\lambda,\theta} \frac{\frac{i_{\text{PD}}(\theta)s(\lambda,\theta)d\lambda \sin\theta\, d\theta}{hc/\lambda}}{\int s(\lambda,\theta)R_{\text{PD}}(\lambda)d\lambda}$$

PD : photodiode

$\lambda : Wavelength\ (WL),\ \Omega :$ Solid angle

$I(\lambda,\theta)$: Radiant intensity at WL reaching the PD (with distance of l) at angle θ

$\Phi(\lambda,\theta)$: Luminous flux at WL reaching the PD (with distance of l) at angle θ

$\Phi_0(\theta)$: Total luminous flux reaching the PD (with distance of l) at angle θ

$s(\lambda,\theta) : (\int s_0(\lambda,\theta)d\lambda = 1)$ = Measured normalized EL spectrum at angle θ

$i_{\text{PD}}(\theta)$: PD current at angle θ

$R_{\text{PD}}(\lambda)$: Responsivity of PD

l = Distance between the PD and the device

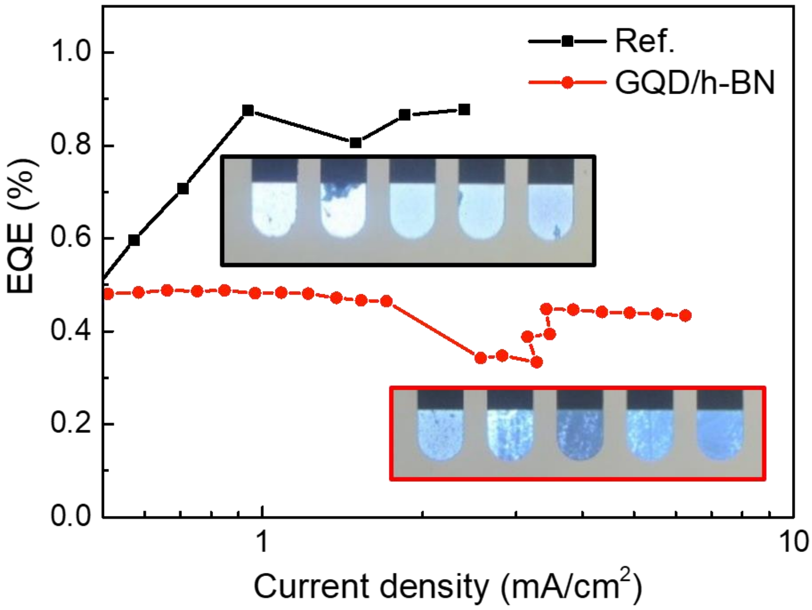

Supplementary Figure 10 | The external quantum efficiency (EQE) of the GQD/h-BN device and reference device.

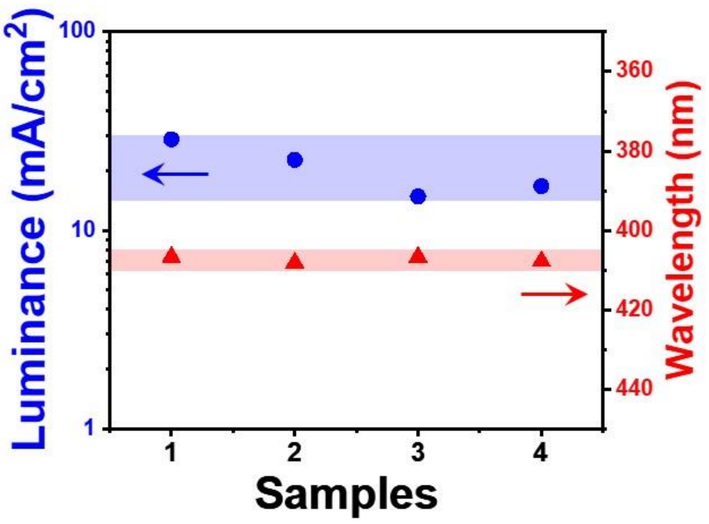

Supplementary Figure 11 | Comparison of EL luminance and wavelength on the different four devices with GQD/h-BN layers. The mean and standard deviation of EL luminance and peak position were 20.75 ± 6.33 and 407.35 ± 0.76 nm, respectively.

References

1. Kim, S.-S. et al. Strain-Assisted Wafer-Scale Nanoperforation of Single-Layer Graphene by Arrayed Pt Nanoparticles. *Chem. Mater.* **27**, 7003-7010 (2015).

2. Kim, G. et al. Catalytic Conversion of Hexagonal Boron Nitride to Graphene for In-Plane Heterostructures. *Nano Lett.* **15** 4769-4775 (2015).

3. Kim, G. et al. Growth of High-Crystalline, Single-Layer Hexagonal Boron Nitride on Recyclable Platinum Foil. *Nano Lett.* **13**, 1834-1839 (2013).

4. Barrios-Vargas, J.E. et al. Electrical and Thermal Transport in Coplanar Polycrystalline Graphene–hBN Heterostructures. *Nano Lett.* **17**,1660-1664 (2017).

5. Weiße, A. et al. The Kernel Polynomial Method. *Reviews of Modern Physics* **78**, 275-306 (2006).

6. Park, M. et al. Efficient Solid-State Photoluminescence of Graphene Quantum Dots Embedded in Boron Oxynitride for AC-Electroluminescent Device. *Adv. Mater.* **30**, 1802951 (2018).

7. Gan, Z., Xu, H. & Fu, Y. Photon Reabsorption and Nonradiative Energy-Transfer-Induced Quenching of Blue Photoluminescence from Aggregated Graphene Quantum Dots. *J. Phys. Chem. C* **120**, 29432-29438 (2016).

8. Song, S.H. et al. Highly Efficient Light-Emitting Diode of Graphene Quantum Dots Fabricated from Graphite Intercalation Compounds. *Adv. Opt. Mater.* **2**, 1016-1023 (2014).

9. Kim, J. K. et al. Origin of White Electroluminescence in Graphene Quantum Dots Embedded Host/Guest Polymer Light Emitting Diodes. *Sci. Rep.* **5**, 11032 (2015).
